# Editorial: Novel insights into the pathophysiology of diabetes-related complications: implications for improved therapeutic strategies, volume II

**DOI:** 10.3389/fendo.2025.1570628

**Published:** 2025-02-27

**Authors:** Jian Ma, Yinghao Guo, Chunjie Jiang, Xuebin Fu

**Affiliations:** ^1^ Department of Immunology, Harbin Medical University, Harbin, China; ^2^ Department of Genomic Medicine, The University of Texas MD Anderson Cancer Center, Houston, TX, United States; ^3^ Department of Cardiovascular-Thoracic Surgery, Northwestern University Feinberg School of Medicine, Chicago, IL, United States; ^4^ Department of Pediatrics, Ann & Robert H. Lurie Children’s Hospital, Chicago, IL, United States

**Keywords:** diabetes, clinical complications, pathophysiology, molecular mechanism, therapeutics

Diabetes mellitus (DM), particularly Type 2 diabetes (T2DM), is one of the most prevalent chronic diseases worldwide, with a wide range of complications that severely affect patients’ quality of life (1–3). Moreover, diabetic complications, such as diabetic retinopathy (DR), diabetic nephropathy (DN), diabetic foot ulcers (DFUs), sarcopenia, and neuropathy, continue to challenge clinical management despite advances in diabetes care (4–6). The mechanisms underlying Diabetes-related complications involve various factors, including metabolic disturbances, immune responses, endothelial dysfunction, and mitochondrial impairment, and so on (7–10). In order to promote a deeper understanding of the pathophysiology of diabetes-related complications, we organized the current Research Topic, “*Novel Insights into the Pathophysiology of Diabetes-related Complications: Implications for Improved Therapeutic Strategies, Volume II*”, following the success of Volume I, which aimed to gather high-quality research that explores these mechanisms.

The Research Topic was initiated on May 23rd, 2023, and closed on Jan 17th, 2025. During these few months, a total of 88 submissions, including 84 manuscripts and 4 abstracts, were received. Finally, 37 high-quality articles were selected and published, covering a wide range of topics related to diabetes-related complications, including DR, DN, diabetic peripheral neuropathy (DPN), T2DM-associated periodontitis, metabolic regulation, immune-inflammatory processes, and emerging biomarkers (Yang et al., Li et al., Li et al., He et al., Xu et al.). These studies have not only provided new insights into the mechanisms driving these complications but also highlighted potential biomarkers, novel diagnostic tools such as branched-chain amino acids (BCAA)(Liu et al.) and immune-inflammatory indices (Li et al., Guo et al.), as well as non-invasive techniques infrared spectroscopy (Zupančič et al.) and single-cell RNA sequencing (Lu et al.). This editorial synthesizes the latest findings based on the accepted papers on this Research Topic, focusing on emerging pathways in the pathogenesis of diabetes-related complications and exploring novel strategies for their prevention and treatment.

## 1 Pathophysiological mechanisms of diabetes-related complications

Studies indicate that a complex interplay of metabolic disturbances, immune responses, and endothelial dysfunction drives the development of diabetes-related complications. These mechanisms represent key targets for developing more effective therapeutic strategies.

### 1.1 Systemic inflammation and immune responses

Inflammation contributes to insulin resistance and microvascular complications such as diabetic kidney disease and diabetic retinopathy (Li et al., Guo et al.). The systemic immune-inflammation index (SII), along with the Neutrophil-to-Lymphocyte Ratio (NLR) and Platelet-to-Lymphocyte Ratio (PLR), has been identified as important biomarkers for assessing the severity of complications like DR and DN (Li et al.). These biomarkers have shown strong associations with the progression of microvascular damage, thus providing early warning signs for at-risk patients and allowing for timely intervention.

### 1.2 Mitochondrial dysfunction and stress response

Mitochondrial dysfunction is central to the pathogenesis of diabetes and its complications. Research highlights the crucial role of Sestrin2 in regulating oxidative stress and mitochondrial function, both of which are vital in the progression of diabetic complications (Zhang et al.). Additionally, disturbances in NAD+ metabolism, particularly through the enzyme KMO, have been implicated in the development of diabetic kidney disease (Yang et al.). The therapeutic potential of mitochondrial transfer therapies, aimed at restoring mitochondrial function, is also emerging as a promising approach to treating metabolic diseases and diabetes-related complications (Chen and Chen). Meanwhile, excitatory neurotransmission and amyloid precursor pathways have also been implicated in diabetes-induced neuropathy. Studies examining VGLUT2 and APP family proteins in STZ-induced diabetes reveal significant neurobiochemical alterations, suggesting that targeting these pathways may offer new strategies for managing diabetic neuropathic changes (Zhang et al.).

### 1.3 Endothelial dysfunction and impaired angiogenesis

Endothelial dysfunction, particularly in diabetic foot ulcers, plays a significant role in delayed wound healing. Through single-cell RNA sequencing, recent studies have revealed that non-healing diabetic foot ulcers exhibit substantial endothelial dysfunction and impaired angiogenesis (Lu et al.). These findings provide important molecular signatures that could be targeted to enhance treatment outcomes for diabetic patients with chronic ulcers and other vascular complications.

### 1.4 Core disturbances in glucose homeostasis and β-cell function

Underlying these complications is the fundamental dysregulation of glucose homeostasis characteristic of diabetes itself (10, Guo et al., Abuhay et al., Habiba et al., Cai et al., Lu et al., Hou et al.). In T2DM, progressive insulin resistance in peripheral tissues combines with β–cell dysfunction, leading to chronic hyperglycemia and compensatory hyperinsulinemia (Guo et al., Diane et al., Cai et al.). Persistent elevations in blood glucose further drive the formation of advanced glycation end–products (AGEs) and exacerbate oxidative stress, which can potentiate inflammatory pathways and vascular injury (Zhang et al., Liang et al.). Over time, diminished β–cell mass and secretory capacity, coupled with heightened insulin resistance, creates a feedback loop that not only worsens metabolic imbalance but also sets the stage for the microvascular and macrovascular complications described above (Roomy et al.).

## 2 Early diagnosis and risk prediction of diabetes–related complications

Timely identification of high–risk individuals is critical for mitigating the severe consequences of diabetes–related complications. Enhanced diagnostic methods and emerging biomarkers pave the way for more targeted intervention and prevention.

### 2.1 Predictive models for diabetic retinopathy and other complications

Given the severe risk of blindness associated with diabetic retinopathy, early detection is essential. Predictive models based on clinical variables such as age, BMI, and HbA1c have been developed to identify patients at high risk of vision–threatening diabetic retinopathy (VTDR) (Gong et al., Kameda). These models enable clinicians to screen high–risk populations effectively, facilitating early interventions to prevent irreversible vision loss. In addition to DR, risk prediction frameworks have been proposed for other acute complications, including diabetic ketoacidosis (DKA), by leveraging large clinical datasets and machine learning approaches for improved accuracy (Liu et al.). Such tools are instrumental in guiding clinicians to tailor monitoring and intervention strategies, ultimately reducing morbidity and mortality in vulnerable groups.

### 2.2 Markers for diabetic kidney disease and other risk indicators

Branched–chain amino acids (BCAAs) have emerged as potential biomarkers for diabetic kidney disease progression (Liu et al.). Elevated BCAA levels correlate with the severity of kidney damage, offering a promising tool for early detection and monitoring of DKD. Beyond kidney disease, additional biomarkers have been linked to various microvascular complications. For instance, systemic immune–inflammation indices (SII), along with the Neutrophil–to–Lymphocyte Ratio (NLR) and Platelet–to–Lymphocyte Ratio (PLR), have shown strong associations with the progression of microvascular damage, thus providing early warning signs for at–risk patients (Li et al., Guo et al.). Moreover, certain circulating inflammatory proteins have been found to correlate with both the onset of Type 2 diabetes mellitus (T2DM) and its complications, potentially serving as early indicators of disease susceptibility (Liang et al.). Furthermore, bioactive leptin (bioleptin) levels have demonstrated utility in gauging metabolic status among children with Type 1 diabetes mellitus, providing a potential avenue for early intervention or tailored therapeutic strategies (Jakubek–Kipa et al.). Integrating such novel biomarkers into existing screening frameworks may refine risk assessment for diverse diabetic complications.

### 2.3 Emerging diagnostic technologies and broader screening approaches

Conventional diagnostic methods have limitations in detecting early stages of diabetes–related complications. Fourier–transform infrared (FTIR) spectroscopy has shown promise as a non–invasive diagnostic tool for assessing skeletal muscle changes in diabetic patients (Zupančič et al.). This technique provides a more efficient way to analyze muscle composition and metabolic changes, serving as an alternative or complement to traditional diagnostic methods. In parallel, physical performance assessments—such as lower extremity function tests—may predict diabetes onset or progression in older adults, indicating that simple functional measures could be integrated into screening protocols (Feng et al.). Furthermore, monitoring for vitamin B12 deficiency, which is highly prevalent in metformin–treated T2DM patients, may help identify individuals at risk of neuropathy or other metabolic derangements (Al Quran et al.). Combining these emerging diagnostic tools with established risk factors and biomarkers holds the potential to refine early detection and guide timely intervention strategies.

## 3 Novel therapeutic strategies for managing diabetes–related complications

Recent insights into the molecular and cellular processes driving diabetes–related complications have prompted the development of innovative interventions. These therapeutic strategies, spanning lifestyle modifications, pharmacological agents, and regenerative approaches, promise more comprehensive and personalized management.

### 3.1 Sarcopenia and diabetes management

Sarcopenia, characterized by muscle loss, is a common complication among elderly diabetic patients. Addressing sarcopenia is essential for improving patient outcomes and preventing further complications. Interventions such as physical exercise, high–protein diets, and pharmacological treatments can help preserve muscle mass and improve metabolic health (Cai et al., Hou et al.). Early detection and intervention can slow the progression of complications such as diabetic nephropathy and diabetic retinopathy. Comprehensive treatment frameworks that integrate both lifestyle and medical management are particularly crucial for elderly patients, given the heightened vulnerability to functional decline and multi–organ impairments.

### 3.2 Pharmacological interventions

New pharmacological treatments offer novel opportunities for managing diabetes and its complications. However, the use of insulin has been linked to an increased risk of diabetic retinopathy (Tan et al.). While other medications, such as GLP–1 receptor agonists and SGLT–2 inhibitors, do not significantly impact DR risk, their role in addressing other aspects of diabetes—such as obesity, cardiovascular health, and glycemic control—warrants further exploration (Roomy et al., Zhang et al.). Some studies have also identified potential therapeutic compounds through bioinformatic and genetic analyses, suggesting drugs like imatinib or topiramate may exert beneficial effects on metabolic pathways (Zhang et al., Zhong et al.). Moreover, stem cell–based therapies, including mesenchymal stem cell (MSC) transplantation, have shown promising outcomes in lowering insulin requirements and improving glycemic control, indicating the broader potential for diabetes care (Habiba et al.).

In the context of acute complications, fluid management remains a cornerstone of diabetic ketoacidosis (DKA) treatment. Recent findings comparing balanced crystalloids and normal saline reveal no significant differences in major clinical outcomes, though balanced crystalloids appear to reduce the incidence of hyperchloremia (Liu et al.). This nuance emphasizes the importance of individualized fluid therapy decisions in DKA management. Ultimately, clinicians must carefully balance each treatment’s risk–benefit profile when designing individualized regimens, considering both chronic and acute facets of diabetes care.

### 3.3 Microbial and immune modulation

The microbiome and immune responses play critical roles in the progression of diabetes–related complications. Studies have highlighted how oral pathogens can influence both diabetes and periodontal disease, suggesting that modulating microbial and immune pathways may offer a novel approach to treating these complications (Li et al.). Parallel findings have demonstrated that systemic immune–inflammation indices, such as SII, NLR, and PLR, may serve as valuable indicators for tailoring anti–inflammatory or immunomodulatory interventions (Li et al., Guo et al.). Emphasizing immune regulation and microbial balance in therapeutic protocols could potentially alleviate complications like chronic infections, periodontal disease, and tissue damage.

### 3.4 Mitochondrial transfer for metabolic diseases

Mitochondrial transfer has emerged as a promising therapeutic strategy for treating metabolic diseases associated with mitochondrial dysfunction. Healthy mitochondria are transferred from donor cells into damaged cells to restore cellular function (Chen and Chen). This approach holds significant potential for treating diabetes–related complications by addressing the underlying cellular dysfunction at the mitochondrial level. As further research explores the feasibility, safety, and scalability of mitochondrial transfer, this method may complement existing pharmacological and lifestyle interventions, contributing to a more holistic management of diabetes and its complications.

## 4 Specific diabetes–related complications and their interactions

Complications such as diabetic retinopathy, nephropathy, and neuropathy often overlap and amplify each other’s impact. Addressing these interconnected pathways holistically may help delay or mitigate multiple complications simultaneously.

### 4.1 Diabetic retinopathy and diabetic nephropathy

There is a growing recognition of the interrelationship between diabetic nephropathy and diabetic retinopathy, with studies indicating that DR may be more severe in patients with nephropathy (Yang et al., Kameda). This highlights the need for integrated treatment strategies that simultaneously address both renal and retinal health in diabetic patients. Attention to factors like long–term glycemic control, inflammation, and oxidative stress remains crucial in preventing the escalation of microvascular damage.

### 4.2 Diabetes and cardiovascular disease

Diabetic patients are at an increased risk for cardiovascular diseases, particularly atherosclerotic cardiovascular disease (ASCVD). Recent research has demonstrated that genes involved in lipid metabolism, such as ABCC5 and WDR7, play key roles in the development of both T2DM and ASCVD (Roomy et al.). Targeting lipid metabolism may offer a dual benefit in managing both diabetes and cardiovascular disease, thus improving overall patient outcomes. In addition, comorbidities such as obesity and dyslipidemia compound ASCVD risk, underscoring the importance of a multifaceted approach to metabolic control (Roomy et al., Zhong et al.).

### 4.3 Other intersecting complications and disease interactions

Beyond DR, DN, and ASCVD, diabetes frequently intersects with other conditions that can exacerbate the disease burden. Research has linked T2DM to acute pancreatitis, gallstone disease, and even autoimmune conditions like primary biliary cholangitis, suggesting shared pathogenic pathways or predispositions (Zhong et al., Lv et al., Yan et al.). Meanwhile, comorbidities such as depression, overactive bladder, and Alzheimer’s disease appear to intensify the progression of diabetic complications, highlighting the importance of holistic patient evaluation (Cai et al., He et al., Ouyang et al.). Furthermore, the severity of diabetic polyneuropathy (DPN) has been shown to be a strong predictor for retinopathy and nephropathy in untreated diabetic patients, underscoring the need for thorough neurological assessments as part of comprehensive risk stratification (Horinouchi et al.). Recognizing these overlapping conditions facilitates integrated management plans tailored to individual patient profiles.

All above published articles on the current Research Topichave provided valuable insights into the pathophysiology of diabetes–related complications, leading to the identification of new biomarkers and therapeutic targets. Emerging strategies such as mitochondrial transfer and immune modulation, as well as novel diagnostic tools like FTIR spectroscopy, hold promise for improving diabetes management and its complications. Targeting key mechanisms like mitochondrial dysfunction, endothelial dysfunction, and systemic inflammation offers new opportunities for personalized therapies that can better address the complex nature of diabetes–related complications.

Future research should continue to validate these biomarkers and treatment strategies through clinical trials and refine predictive models for earlier intervention. Multidisciplinary collaboration, incorporating advances in molecular biology, clinical research, and technological advances, will be essential for developing more effective therapeutic approaches for diabetes and its associated complications. This editorial aims to inspire further studies and collaborations to enhance the lives of the millions affected by diabetes worldwide, ultimately leading to more efficient prevention and treatment of its far–reaching complications.
